# Cone-beam computed tomographic evaluation of styloid process: a retrospective study of 208 patients with orofacial pain

**DOI:** 10.1186/1746-160X-10-5

**Published:** 2014-02-15

**Authors:** Haluk Öztunç, Burcu Evlice, Ufuk Tatli, Ahmet Evlice

**Affiliations:** 1 Department of Oral and Maxillofacial Radiology, Faculty of Dentistry, Çukurova University, Saricam, 01330 Adana, Turkey; 2 Department of Oral and Maxillofacial Surgery, Faculty of Dentistry, Çukurova University, Adana, Turkey; 3 Department of Neurology, Faculty of Medicine, Çukurova University, Adana, Turkey

**Keywords:** Cone-beam computed tomography, Eagle’s syndrome, Elongated styloid process, Orofacial pain, Retrospective study

## Abstract

**Introduction:**

The purpose of this study was to assess the structural characteristics of styloid process (SP) by cone-beam computed tomography (CBCT) examination in a patient population suffering from orofacial pain. The second aim was to assess the prevalence of elongated SP and its relation to gender, site and subjective symptoms in the study population.

**Methods:**

Clinical and radiographic records of 208 patients were evaluated retrospectively. Radiological examinations including measurements of the structure, length, and medial angulations of SP were performed on CBCT images.

**Results:**

Out of 208 patients, 96 (46%) had not-elongated SP, 28 (13%) had left side, 16 (8%) had right side, and 68 (33%) had bilateral elongation of SP. The patients with elongated SP had significantly decreased angle values. There were no statistically significant differences in length values of SP between males and females in both groups. Significantly increased prevalence of symptoms except headache was observed in patients with elongated SP.

**Conclusions:**

This study presents the CBCT as an alternative method to CT or panoramic radiographs for the measurement and the assessment of the styloid process. Patients suffering from orofacial pain, who also had elongated SP, had increased rate of corresponding neurological complaints compared with non-elongated ones.

## Introduction

Eagle’s syndrome (ES) is characterized by recurrent pain in the oropharynx and face secondary to calcification of the stylohyoid ligament (SHL) or elongated styloid processes (ESP) greater than 30 mm [1]. ES can occur unilaterally or bilaterally and frequently results in symptoms of dysphagia, recurrent throat pain and foreign object sensation, referred otalgia, headache, pain on rotation of the neck, dizziness, pain on extension of the tongue, pain on opening mouth, discomfort during chewing, change in voice, and a sensation of hypersalivation [2]. Diagnosis is guided both by clinical and radiologic examination. Palpation of the styloid process (SP) in the tonsillar fossa is indicative of elongation [2]. X-rays are used to diagnose ESP; panoramic radiography (OPG) and computed tomography (CT) of the skull base and neck are the preferred radiographic studies [3].

However, in the recent past, three-dimensional (3-D) cone-beam computerized tomography (CBCT) which can definitively measure the length of the anatomical structures of craniofacial region is introduced as a new and alternative modality [4]. CBCT allows images to be acquired with a low dose of radiation, shorter patient examination time and lower costs than conventional CT, which make its routine use practicable for oral and maxillofacial imaging and surgical procedures. This recently-designed technology has become a relevant tool for diagnostic imaging of oral and maxillofacial osseous structures, providing to professionals access to excellent image quality and greater diagnostic accuracy and sensitivity [4].

The treatment of ES is primarily surgical through an intraoral or extraoral approach [5,6]. Nonsurgical treatments include reassurance, non-steroidal anti-inflammatory medications, analgesics, anticonvulsants, antidepressants and local infiltrations with steroids or anesthetic agents [3]. Patients who fail medical therapy may benefit from surgical removal of the elongated portion of the SP.

Although there are numerous reports on SP length and ES using panoramic radiograph or CT, we could not find any study based on CBCT examination to evaluate the length and medial angulations of SP and related clinical complaints which should be differentiated from neurological situations such as glossopharyngeal neuralgia, recurrent or persistent head and neck pain, and dizziness.

The purpose of the present study was to assess the structural characteristics of SP by CBCT examination in a patient population suffering from orofacial pain. The second aim was to assess the prevalence of elongated SP and its relation to gender, site and subjective symptoms in the study population.

## Materials and methods

The clinical and radiographic records of 208 patients suffering from neurological symptoms in maxillofacial region who had referred to neurology department and then consulted to the faculty of dentistry for CBCT examination, during the period from January 2011 to January 2013, were evaluated retrospectively. The study’s protocol was carried out according to the principles described in the Declaration of Helsinki, including all amendments and revisions. Due to the retrospective nature, the present study is exempt in accord with the institutional review board (Ethical Review Board of Çukurova University Medical Scientific Researches) standards of our institution.

Clinical symptoms including pain and all kind of discomfort in the maxillofacial and neck region including dizziness, tinnitus, otalgia, dysphagia, foreign body sensation, and pain on turning head were recorded from the patients’ charts by neurologist author. When ES was suspected as an initial diagnosis during neurological examination due to the corresponding complaints of the patients, the physician consulted the patients to the maxillofacial radiology department for CBCT imaging in order to differentiate the diagnosis. This was the patient population included for the present study. As well as clinical symptoms, the radiological examination of the structure, length, and medial angulations of SP were performed on CBCT (Iluma Cone Beam CT Scanner, Imtec Imaging, LLC, Ardmore, OK, USA) images of the patients in order to investigate the incidence and characteristics of ES in this patient population.

**Figure 1:**
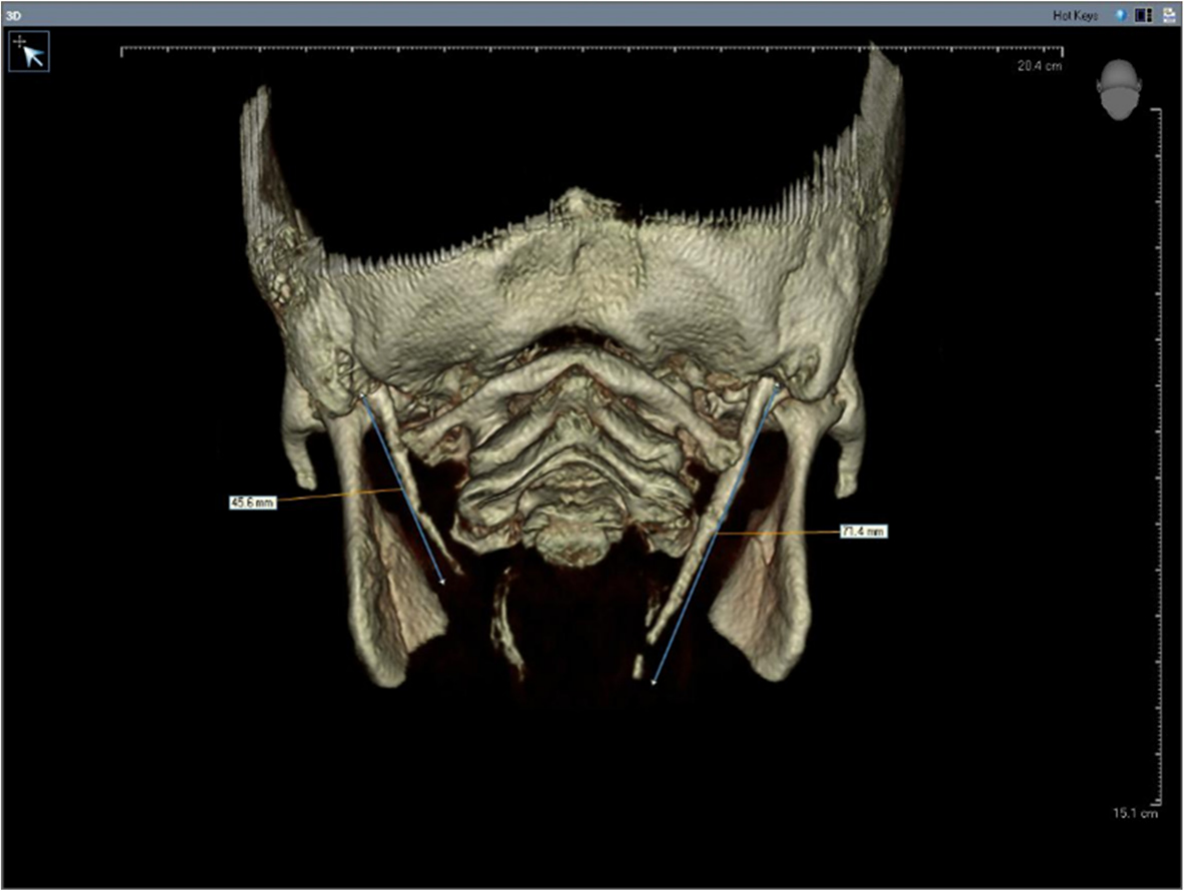
The measurement of the styloid process length.

**Figure 2:**
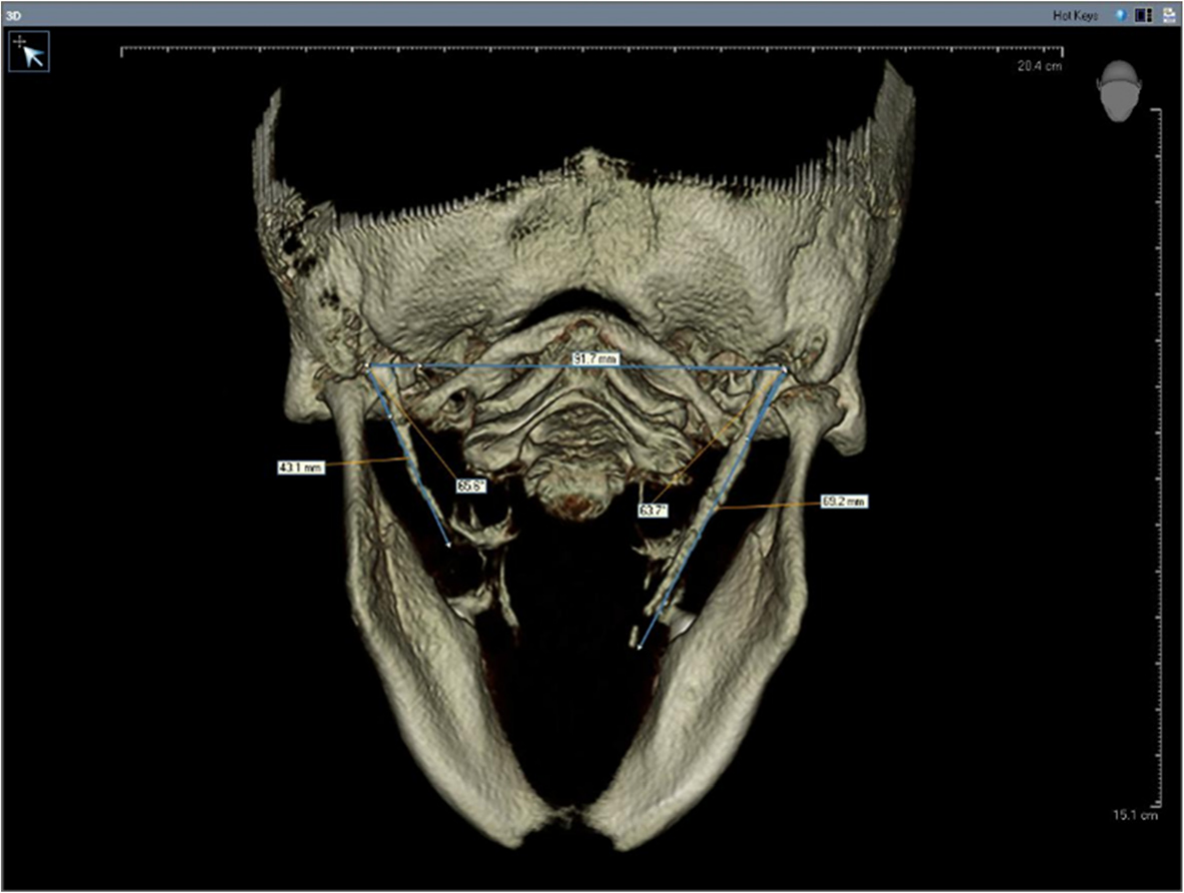
The measurement of the angle between the line connecting the base of the both styloid processes and the axis of the styloid process.

All the CBCT examinations had been taken using 120 kV, 3,8 mA and 20 seconds exposure time and by positioning the reference points on the face of the patients (centre line, frankfurt horizontal plane, and condyle guide light) recommended by the producing company. Only high-quality scans were included. Images of low quality, such as scattering or insufficient accuracy of bony borders were excluded. Radiological measurements of 416 SPs were performed on CBCT images of the 208 patients by two oral and maxillofacial radiologists. If they did different measurements, they discussed until agree with each other. The length and angle values were recorded separately for each side since there were some differences between the right and left sides in some cases. Styloid processes were evaluated for their average lengths and angles. The length of SP was measured from the caudal margin on the tympanic pleat to the tip of the process (Figure 1). If the cranial part of the SP was not visible, the length between the probable attachment point to the calvaria and the tip of the SP was measured. The ossification of SHL that joined SP was added to the measurement. The angle between the line connecting the base of the both SPs and the axis of the SP was also measured (Figure 2).

The general structural appearances of SP and calcifications and ossifications of SHL were also evaluated according to the system of Langlais et al. [7] The morphology of the stylohyoid complex was classified into three types: Type 1 represents an uninterrupted, elongated SP. Type 2 is characterized by the SP apparently being joined to the SHL by a single pseudoarticulation. Type 3 consists of interrupted segments of the mineralized ligament, creating the appearence of multiple pseudoarticulations within the ligament. Calcification patterns of SP were classified into four types: Pattern A (calcified outline) describes a thin radiopaque border with a central radiolucency that constitutes the majority of the process. Pattern B (partially calcified) describes a process that has a thicker radiopaque outline and complete opacification but small, sometimes discontinuous, radiolucent cores. Pattern C (nodular) has a knobby or scalloped outline. It may be partially or completely calcified with varying degrees of central radiolucency. Pattern D (completely calcified) is totally radiopaque with no evidence of a radiolucent interior.

### Statistical analysis

Patients were grouped according to gender, elongation of SP, and calcification type and pattern of SP. The appropriate method to test the statistical significance of difference in a classification system was Chi-square test. So, Chi-square test was used to assess the prevalence of the calcification type and pattern of SP among site and gender. Due to the number of the patients and normal distribution of the data, a parametric test, independent sample t-test, was used to compare the mean age of the patients, and mean angle and length of SP according to gender and elongation status. The appropriate method to calculate the ratio of the odds of the outcome in two groups in retrospective studies was Odds ratio test. So, Odds ratio test (OR) was used to compare the prevalence and the ratio of the odds of the subjective symptoms in patients with and without elongated SP. Statistical analysis was performed using MedCalc software (version 10.1.6, Mariakerke, Belgium). P < 0.05 was considered significant.

## Results

**Figure 3:**
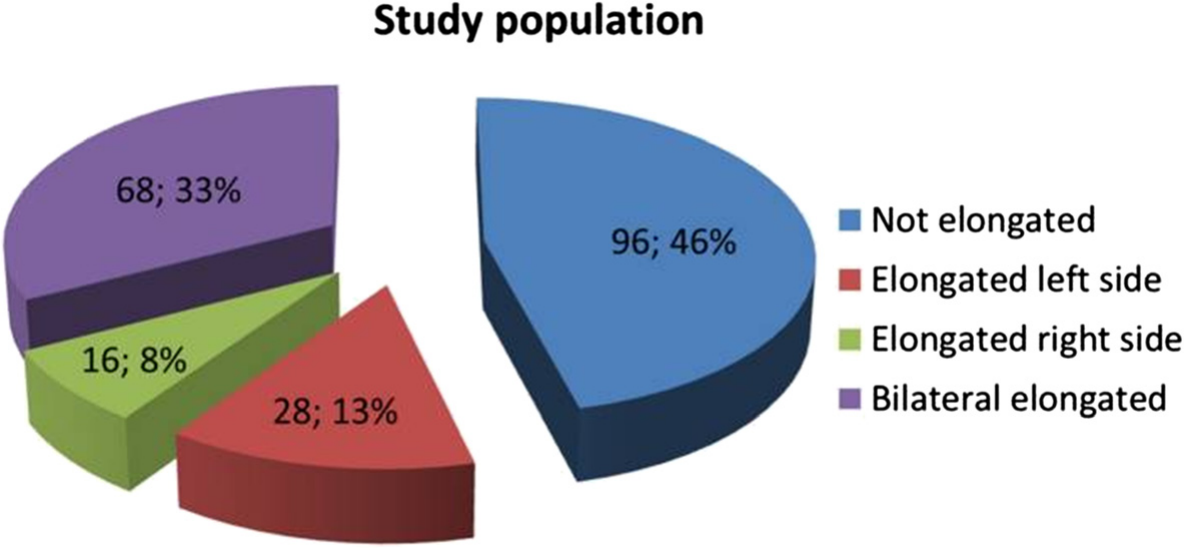
The distribution of the study population according to styloid process elongation.

Out of a total of 208 patients, 96 subject (46%) had not-elongated SP, 28 (13%) had left side, 16 (8%) had right side, and 68 (33%) had bilateral elongation of the SP (Figure 3). Absence of proximal part of the SP was detected in 5 cases unilaterally and in 4 cases bilaterally. SP was not detected in 3 cases bilaterally and in 6 cases unilaterally.

The types of SP and the patterns of calcification stratified according to gender are listed in Table 1. Type 2 was the most frequent type of SP encountered irrespective of laterality (212/416). The most frequent pattern of calcification was “partially calcified (Pattern B)” irrespective of laterality (156/416). No statistically significant differences were proven between the types of SP calcification among the gender and site prevalence (Chi-square test) (P > 0.05). In terms of the patterns of SP calcification, statistically significant difference was only found in Pattern C among gender and site prevalence (Chi-square test) (P = 0.011) (Table 1). The prevalence of Pattern C styloid process in males was significantly increased in the right site. However, it was significantly increased in the left site in females. The relationship between gender and elongation of the styloid process was not statistically significant (Chi-square test) (P = 0.771).

**Table 1:** Comparison of the calcification type and pattern of styloid process among site and gender

**Styloid**	**Site**	**Male**	**Female**	**x**^**2**^	**p**
**Calcification**					
Calcification type					
Type 1	Right	36	32	0.699	0.403
	Left	32	40		
Type 2	Right	56	48	0.431	0.511
	Left	64	44		
Type 3	Right	24	12	0.018	0.891
	Left	20	8		
Calcification pattern					
Pattern A	Right	44	16	0.409	0.522
	Left	48	24		
Pattern B	Right	40	44	0.018	0.891
	Left	36	36		
Pattern C	Right	8	4	6.328	0.011
	Left	0	8		
Pattern D	Right	24	28	0.901	0.342
	Left	32	24		

The mean age of the patients with elongated SP (47.28 ± 12.4 years) was lower than those without elongated SP (50.54 ± 13.01 years) but the difference was not statistically significant (Independent sample t-test) (p = 0.066). The patients with elongated SP had significantly decreased angle values compared with non-elongated ones (Independent sample t-test) (P < 0.001) (Table 2). In terms of gender characteristics, males had significantly higher angle values than females in both elongated (P < 0.001) and non-elongated (P = 0.001) groups (Independent sample t-test) (Table 2). There were no statistically significant differences in length values of SP between males and females in both elongated (P = 0.157) and non-elongated groups (P = 0.387) (Independent sample t-test) (Table 3).

**Table 2:** Comparison of the difference in mean angle of the styloid process according to gender and elongation status

**Patients**	**Elongated**		**Not elongated**		**p value**
	**N**	**Angle (Mean ± SD)**	**N**	**Angle (Mean ± SD)**	
Male	64	69.58 ± 3.63°	52	71.25 ± 3.77°	0.017
Female	48	66.19 ± 2.99°	44	68.54 ± 4.06°	0.002
Total	112	68.13 ± 3.76°	96	70.01 ± 4.12°	0.000

**Table 3:** Comparison of the difference in mean length (cm) of the styloid process according to gender

**Patients**	**Male**		**Female**		**p value**
	**N**	**Length (Mean ± SD)**	**N**	**Length (Mean ± SD)**	
Elongated	64	3.62 ± 0.48 cm	48	3.81 ± 0.84 cm	0.157
Not elongated	52	2.02 ± 0.56 cm	44	2.11 ± 0.42 cm	0.387
Total	116	2.90 ± 0.95 cm	92	3 ± 1.09 cm	0.495

In terms of subjective symptoms, significantly increased prevalence of symptoms except headache was observed in patients with elongated SP (P < 0.05) (Odds ratio) (Table 4).

**Table 4:** Comparison of the prevalence of the subjective symptoms between patients with and without elongated styloid process

**Symptoms**	**Elongated**	**Not elongated**	**OR**	**95% CI**	**p**
	**N (%)**	**N (%)**			
Headache	88 (78.6)	84 (87.5)	0.5	0.2 – 1.1	0.093
Dizziness	44 (39.3%)	24 (25%)	1.9	1.1 – 3.5	0.029
Tinnitus	16 (14.3)	4 (4.2)	3.8	1.2 – 11.9	0.020
Otalgia	20 (17.8)	4 (4.2)	5	1.6 – 15.2	0.004
Dysphagia	68 (60.7)	8 (8.3)	17	7.5 – 38.5	0.000
Foreign body sensation	40 (35.7)	3 (3.1)	17.2	5.1 – 57.9	0.000
Pain on turning head	59 (52.7)	5 (5.2)	20.3	7.6 – 53.6	0.000

*Abbreviations*: *OR* Odds ratio, *CI* confidence interval.

## Discussion

In the present study, the incidence and characteristics of ESP among the patients suffering from orofacial pain who had been referred for CBCT imaging were evaluated retrospectively. It is important to be aware of presence of SP elongation for all health care professionals deal with the diagnosis and treatment of head and neck pain. The apex of the SP is clinically important because it is located between internal and external carotid arteries, just lateral to the tonsillar fossa within the lateral pharyngeal wall. The SP is also related with facial nerve anteromedially, and accessory and vagus nerves medially [8].

The patients having ES have often been treated by family physicians, otolaryngologists, neurologists, neurosurgeons, dentists, maxillofacial surgeons, and psychiatrists. However success is very little, because clinicians frequently fail to diagnose ES. The misdiagnosed patients with ES may undergo unnecessary treatments and surgical procedures [6]. Thus, a correct differential diagnosis is crucial to distinguish elongated SP from other situations with partially overlapping symptoms. The differential diagnosis for ES includes cervical myofacial pain syndrome, migraine, trigeminal neuralgia, glossopharyngeal neuralgia, nervus intermedius neuralgia, nasopharyngeal mass/lesion, tonsillitis, otitis, degenerative diseases causing neck pain, psychosomatic diseases, vascular compromise (atherosclerosis), pain of dental origin, and TMJ problems [6,9]. In case of head and face pain refractory to treatment or unexplained neurologic complaints of head and neck region, ES could be considered in the differential diagnosis.

The incidence of the ESP is controversial (ranges between 1.4% and 30%) in the literature [2,6,8]. The incidence of the ES is much lower than the incidence of ESP. Only small percentages (between 1% and 5%) of the patients were reported to actually be symptomatic [7,10]. The results of the present study showed that 54% of the study population had ESP which could contribute the complaints of the patients. The higher incidence rate observed in the present study might be due to the special study population suffering from orofacial pain. The results also revealed that ESP seems to be related with the appearance of certain neurological symptoms presented in Table 4.

Clinical symptoms of ES are related to the size of stylohyoid complex [8]. Therefore, the full length of SP should be visualized for its measurement. On the basis of size, SP is divided into two types, normal and elongated. The threshold for elongation is highly variable but 30 mm were considered as the threshold by many publications [8,11,12].

Most frequently, a panoramic radiograph is used to determine whether the SP is elongated [8,13]. Many factors, such as magnification of the different panoramic machines, the angle between the stylohyoid complex and the cranium base can affect the apparent length of the stylohyoid complex in the panoramic radiographs [8]. Accurate determination using 2-D radiographic examinations is difficult due to projection geometry considerations and superimpositions of mandibula and teeth on SP. In terms of metric assessment, panoramic radiographic techniques may cause distortion of the length and angulation of the styloid process [13]. Thus advanced imaging techniques are required to overcome this issue. In 3-D CT reconstruction, there is no geometric error due to magnification effects. The true character of bilaterally asymmetrical malformations may be evaluated, since overlap of structures is not encountered. Operator-related error is also minimized in 3-D CT [14]. But 3-D CT imaging has some limitations such as a slight movement may result in degradation of images and higher radiation dose is required, depending upon the number of sections taken [14]. CBCT may be recommended as a dose-sparing technique compared with standard medical CT scans for common oral and maxillofacial radiographic imaging tasks [15,16]. The advantages of CBCT imaging are the following: lower radiation dose than conventional CT, the possibility of individualized overlap-free reconstructions, and DICOM data can be imported and exported for other applications [17]. In the literature, panoramic radiograph and CT was used as a diagnostic tool in most of the studies evaluating the structural characteristics of elongated SP [13,14,18]. Up to our knowledge, there is no study based on CBCT examination for the evaluation of structural characteristics of elongated SP in the literature.

Anbiaee and Javadzadeh [8] used panoramic radiograph for the measurement of SP length and indicated that SP length was associated with increasing age. In the present CBCT examination, we did not find a relationship between patient age and SP length in overall study population. However, female patients with not-elongated SP were significantly older than those with elongated SP (P < 0.001). The difference about this issue in different studies may be due to the race of the patients and diagnostic machines used for measurement.

In the present study, the average length of SP between male and female patients was not significantly different neither in elongated nor not-elongated group, indicating no relationship between gender and length of SP, similar to previous studies [18,19].

The most common morphology of SP was pseudoarticulated type in both male and female patients in the present study. On the contrary Kursoglu et al. [13] and Anbiaee and Javadzadeh [8] indicated continuous type as the most common type of morphology in their studies in which OPG was used as diagnostic tool. In the present study, the most common pattern of ossification was partially calcified similar to previous study from Turkey [20].

The prevalence of elongated and symptomatic SP is not exactly known since different length values were reported in the studies. The reason for different variations in the measurements of SP in various studies could be the difference in imaging methods. The assessment of the length of SP might be effected by the magnification of the panoramic devices and by the angulations of the SP in 2D imaging technology. Moreover, symptoms also depend on the angulation of SP as well as length [18]. These parameters can only be measured by advanced imaging techniques exactly. When the angle of SP is narrow, it may be supposed to produce some complaints due to compression of adjacent structures. The present study revealed that patients with elongated SP had significantly narrow angle value than those with not-elongated SP (P < 0.001). Similary, female patients had significantly narrower angle values than male patients in both elongated and not-elongated groups (P < 0.05) which could make female patients more prone to corresponding complaints. It was also observed that prevalence of subjective symptoms was significantly higher in the patient population with elongated SP. Nayak et al. [14] reported similar symptoms in patients with elongated SP. Moreover, a significant correlation between the angle and length values of SP was observed in the present study (Pearson’s correlation) (r = −0.229, P < 0.001).

## Conclusions

This study presents the CBCT as an alternative method to CT or panoramic radiographs for the measurement and the assessment of the styloid process. Within the limitations of the present retrospective study in a special patient population; it was observed that the patients suffering from orofacial pain, who also had ESP, had increased rate of corresponding neurological complaints compared with non-elongated ones. Further clinical studies, also including patients with ESP but without pain complaints, is necessary to evaluate the exact correlation between presence of styloid elongation and neurological complaints.

## Abbreviations

SP: Styloid process; CBCT: Cone-beam computed tomography; OPG: Panoramic radiography; ES: Eagle’s syndrome; SHL: Stylohyoid ligament; ESP: Elongated styloid process; CT: Computed tomography; 3-D: Three-dimensional.

## Competing interests

The authors declare that they have no competing interests.

## Authors’ contributions

HÖ, BKE, UT and ATE collected the data. HÖ and BKE performed the measurements of the subjects. UT analysed and interpreted the data. The concept of the paper was devised by UT and ATE. BKE and UT drafted and wrote the manuscript. All authors read and approved the final manuscript.
